# Exosome-based *Sfrp2* inhibition in mesangial cells alleviates osteoporosis and promotes osteointegration in diabetic kidney disease

**DOI:** 10.1093/rb/rbaf093

**Published:** 2025-09-02

**Authors:** Helin Xing, Yang Liu, Mi Qu, Zhengping Zhang, Yuhong Zeng, Pan Li, Qingsong Jiang, Guodong Yang

**Affiliations:** Department of Prosthodontics, Beijing Stomatological Hospital and School of Stomatology, Capital Medical University, Beijing 100050, China; Department of Ultrasound Diagnostics, Tangdu Hospital, Fourth Military Medical University, Xi’an 710038, China; Department of Ultrasound Diagnostics, Tangdu Hospital, Fourth Military Medical University, Xi’an 710038, China; Department of Biochemistry and Molecular Biology, School of Basic Medicine, The Fourth Military Medical University, Xi’an 710032, China; Department of Spine Surgery, Honghui Hospital, Xi’an Jiaotong University, Xi’an 710054, China; Department of Spine Surgery, Honghui Hospital, Xi’an Jiaotong University, Xi’an 710054, China; Department of Orthopedic Surgery, Xijing Hospital, The Fourth Military Medical University, Xi’an 710032, China; Department of Prosthodontics, Beijing Stomatological Hospital and School of Stomatology, Capital Medical University, Beijing 100050, China; Department of Biochemistry and Molecular Biology, School of Basic Medicine, The Fourth Military Medical University, Xi’an 710032, China

**Keywords:** diabetic kidney disease, osteoporosis, single-cell sequencing, SFRP2, exosomes

## Abstract

Diabetic kidney disease (DKD) and osteoporosis are closely linked, yet the underlying mechanisms remain incompletely understood. DKD mouse and rat models were established via combinatorial treatment with a high-fat diet and streptozotocin, which not only induced progressive renal dysfunction, but also triggered systemic osteoporotic changes, including reduced bone mineral density, trabecular thinning and impaired bone microarchitecture. Using single-cell sequencing, we demonstrate that DKD elevates the expression of *Sfrp2* (secreted frizzled related protein 2) in glomerular mesangial cells (MCs), establishing MCs as a critical source of circulating secreted frizzled related protein 2 (SFRP2 protein). In turn, elevated SFRP2 potently inhibits the Wnt signaling pathway, suppresses osteoblast differentiation and promotes bone loss in diabetic mice. Exosomes, which exhibit a size range endowed with natural tropism for the renal mesangial space, hold promise as optimal delivery vectors targeting renal MCs. Exosomes loaded with *siSfrp2* (siRNA against *Sfrp2* mRNA) circulate into MCs after tail vein injection. In turn, exosome-mediated *siSfrp2* delivery effectively reduces circulating SFRP2 levels, restores Wnt signaling and alleviates osteoporotic phenotypes in DKD mice. Moreover, in diabetic rat models, renal injury is accompanied by consistent osteoporotic defects and weakened implant osteointegration capacity. Exosome-mediated *Sfrp2* knockdown in these rats significantly enhances implant osseointegration, further validating the renal-osteal axis. These findings establish a MCs-derived SFRP2-mediated renal-osteal axis, revealing that glomerular MC-secreted SFRP2 serves as a key molecular bridge linking kidney injury to bone loss. This mechanistic insight highlights SFRP2 and its main cellular source (MCs) as promising therapeutic targets for managing diabetic osteoporosis.

## Graphical abstract

**Figure:**
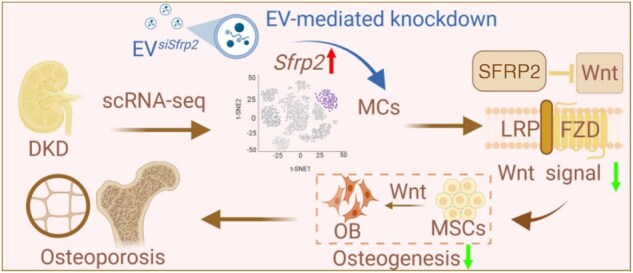

## Introduction

Diabetes mellitus (DM) is a global health challenge. Diabetic kidney disease (DKD) and osteoporosis are two prevalent complications among diabetic patients [1], often co-existing and exacerbating each other’s pathological processes [2–4]. This crosstalk is mediated by renal-derived factors such as Klotho, FGF23, erythropoietin (EPO) and extracellular vesicles (EVs), which regulate calcium-phosphate homeostasis and osteoblast/osteoclast activity [5–7]. However, the intricate molecular mechanisms connecting these two conditions remain elusive.

The Wnt signaling pathway plays a crucial role in bone homeostasis, regulating osteoblast differentiation and function [8]. Recently, renal MCs derived EVs containing Wnt pathway regulators under diabetic conditions were found to play an important role in osteoporosis. Among the Wnt pathway regulators, SFRP2, as an antagonist of the Wnt signaling pathway, has been shown to inhibit osteoblastogenesis and bone formation [9–12]. In the context of DKD, increased SFRP2 has been revealed, while the role of SFRP2 and its potential impact on bone metabolism are largely unknown [13].

Recent advancements in single-cell sequencing technology have enabled in-depth exploration of cellular heterogeneity and molecular mechanisms in complex diseases [14, 15]. Meanwhile, exosomes, small extracellular vesicles secreted by cells, have attracted extensive attention in the research community. Exosomes can carry various bioactive molecules, such as proteins, nucleic acids and lipids, and play a key role in cell-to-cell communication. Their high biocompatibility, low immunogenicity and ability to cross biological barriers make them ideal carriers for therapeutic agents [16–19]. In the field of disease treatment, exosome-mediated delivery of nucleic acids has emerged as a promising strategy for targeted therapy [20, 21]. This makes exosomes a potential tool to explore the interorgan crosstalk and new therapeutic strategies. Moreover, nanoparticle size is a key determinant for deposition in the mesangial space [22–24], and exosomes are endowed with natural tropism for the renal mesangial space due to their size range, holding promise as optimal delivery vectors targeting renal MCs.

While DKD is associated with reduced bone density through mechanisms like Klotho deficiency, dysregulated Wnt and TGFβ signals [25–27], whether SFRP2-mediated Wnt inhibition represents novel renal-bone crosstalk remains unaddressed. Here, we integrate single-cell profiling of diabetic kidney and exosome-based functional intervention to test the hypothesis that MC-derived SFRP2 exacerbates osteoporosis, and reducing SFRP2 expression could serve as an effective therapeutic approach.

## Materials and methods

### Animal treatment

All animal experiments were approved by the Animal Care and Use Committee of Fourth Military Medical University. Six-week-old C57BL/6 mice (weighing 20–25 g) were obtained from the Laboratory Animal Center of Fourth Military Medical University. The mice were housed in a controlled environment with regulated temperature and humidity, and they had unrestricted access to food and water. After a one-week acclimatization period, the mice were given a high-fat diet for four weeks. Subsequently, the mice in the high-fat diet group received intraperitoneal injections of streptozotocin (STZ) (Beyotime, dissolved in 0.1 M sodium citrate buffer, pH 4.5) at a dose of 50 mg/kg/day for five consecutive days. The control mice were injected with an equivalent volume of sodium citrate buffer. A tail vein blood glucose concentration exceeding 16.7 mM one week after STZ injection confirmed the diagnosis of DM. For the induction of diabetes in rats, male Sprague-Dawley rats weighing 200–230 g were acclimatized for one week under standard laboratory conditions with free access to standard rodent chow and water. The rats were then randomly assigned to either the control group or the STZ-induced diabetic group. Freshly prepared STZ in 0.1 M citrate buffer (pH 4.5) at a concentration of 60 mg/kg body weight was administered to the diabetic group after an overnight fast for five consecutive days. Both mice and rats were maintained for an additional eight weeks to induce kidney injury.

### Urinary albumin-creatinine ratio assay

The urine samples from the mice were collected using a metabolic cage over a 12-h period for four weeks and stored at −80°C. The urinary albumin levels were quantified using a Mouse Albumin ELISA Quantification Kit (E-EL-M0792c, Elabscience, Wuhan, China), while urine creatinine levels were measured with a Creatinine Assay Kit (CO11-2-1, Jiancheng, Nanjing, Jiangsu, China) [28].

### Kidney histological analysis

The kidney tissues were fixed in 4% paraformaldehyde for 24 h at 4°C, after which they were transferred to PBS containing 30% sucrose for an additional 24 h. The tissues were then embedded in optimal cutting temperature (OCT) compound and sectioned to a thickness of 8 µm. The sections were subjected to Hematoxylin-Eosin (H&E), Masson’s trichrome and Periodic Acid-Schiff (PAS) staining. Quantitative analysis was conducted using ImageJ software.

### Chromium 10 ×  genomics library construction and sequencing

The kidney tissues were collected from mice and stored in MACS^®^ Tissue Storage Solution at 4°C. The tissues were dissociated into single-cell suspensions using standard dissociation protocols, followed by the lysis of remaining red blood cells and the removal of dead cells. The library was sequenced on an Illumina 6000 sequencing system (paired-end multiplexing run, 150 bp) by LC-Bio Technology (Hangzhou, China) at a minimum depth of 20 000 reads per cell [29].

### Exosome isolation, characterization, loading and tracing

HEK293 cells were cultured in exosome-depleted medium for 48 h, after which the supernatant was collected exosome isolation. The culture medium was first centrifuged for 15 min to remove cell debris. The supernatant was then filtered through 0.22 μm filters and pelleted by centrifugation. Exosomes were resuspended in PBS and stored at −80°C until further use.

Isolated exosomes were further validated using Nanoparticle Tracking Analysis, transmission electron microscopy (TEM) and Western blotting. To load siRNA into exosomes, siRNA (20 μM) was combined with 1 × 10^10^ exosomes and suspended in 400 μl, and then, electroporated at 700 V/150 mF using the Gene Pulser Xcell™ Electroporation System (Bio-Rad). Free siRNA was removed through additional exosome isolation via ultracentrifugation. Loading efficiency was calculated as (1 - free siRNA concentration/original concentration) × 100%. The detailed sequences of the siRNAs used are listed in Supplementary Table S1. For *in vivo* tracking of exosome distribution, exosomes were first incubated with the fluorescent dyes DiR/DiI at a final concentration of 10 μM. Labeled exosomes were then injected into mice or rats via the tail vein. Exosome distribution in the indicated organs was analyzed using *ex vivo* imaging or confocal microscopy.

### Exosome therapy

*siSfrp2* loaded exosomes were injected into diabetic mice or rats via tail vein once a week for 8 weeks at 4 mg/kg body weight. Exosomes loaded with siNC (negative control siRNA) served as control.

### Surgical procedure of implantation

The rats were anesthetized intraperitoneally with 1% pentobarbital (0.4 mL/100 g) and received local anesthesia with prilocaine (Pierre Rolland, Bordeaux, France) to ensure surgical comfort. A 1.5 cm lateral incision was made approximately 1 cm proximal to the distal femoral condyles to expose the underlying bone. A 1.0 mm diameter hole was then drilled into the metaphyseal region of the distal femur for the placement of a titanium implant (1.5 mm × 5 mm). During drilling, iced saline irrigation was employed to prevent overheating and to ensure optimal conditions for implant placement. After the implant was securely inserted, the wound was closed in layers with sutures. Postoperatively, the rats were housed individually and allowed to move freely in their cages. They were provided with standard laboratory chow and water *ad libitum*. Antibiotics were administered as needed to prevent infection. The rats were monitored daily for any signs of infection or abnormal behavior. At predetermined time points (4, 8 and 12 weeks after implantation), the rats were sacrificed under anesthesia. The femurs containing the implants were carefully harvested and processed for further analysis.

### Methylene blue/acid fuchsin staining

For histological analysis of the rat femur bone-implant interface, femurs were fixed in 4% paraformaldehyde at 4°C for 24 h to preserve tissue structure. Following fixation, the samples were dehydrated through a graded series of ethanol (ranging from 70% to 100%) and embedded in resin using the Technovit^®^ 9100 Kit (EMS). The specimens were sectioned into 50 μm slices without decalcification. The sections were stained with a combination of methylene blue and acid fuchsin. Specifically, the sections were first stained with acid fuchsin, which binds to the bone matrix and imparts a pinkish-red color. Subsequently, the sections were treated with an acid-alcohol solution (3% HCl in 95% ethanol) to remove non-specific staining. Finally, the sections were counterstained with methylene blue, which highlights the cellular components and any remaining unstained areas in blue. Digital images of the stained sections were captured using a light microscope (Olympus, Tokyo, Japan).

### Enzyme-linked immunosorbent assay

Serum and kidney SFRP2 levels were analyzed by Enzyme-Linked Immunosorbent Assay (ELISA) (Mouse SFRP2 from ELK Biotechnology and Rat SFRP2 from Zeye Biotechnology). The tests were performed according to the manufacturers’ instructions.

### RNA isolation and qPCR assays

Total RNA from cells and tissues was isolated using Trizol Isolation Reagent (Roche, USA) according to the manufacturer’s instructions. Quantitative PCR (qPCR) reactions were conducted using FastStart Essential DNA Green Master (Roche, Basel, Switzerland). All PCR reactions were performed in triplicate, with GAPDH serving as a normalized control for target RNA expression. Relative expression levels were calculated by normalizing to the control samples using the 2^-ΔΔCt method. The sequences of the PCR primers are provided in Supplementary Table S2.

### Western blot analysis

Cell, exosome and tissue protein samples were prepared using RIPA lysis buffer and protein quantification was performed using the BCA method. The samples were separated by electrophoresis on 10% SDS-PAGE gels at 80–120 V. Proteins were transferred to a nitrocellulose membrane at 200 mA for 2 h, blocked with 5% bovine serum albumin (BSA) for 2 h and incubated overnight at 4°C with primary antibodies (GM130, CD9, TSG101, SFRP2, GAPDH). The membrane was washed with TBST and then incubated with the corresponding secondary antibodies at room temperature for 1 h. Finally, the membranes were detected using an enhanced chemiluminescence (ECL) detection reagent (Mishu Biotechnology, Xi’an, China), and the signal intensities were quantified using a bio-imaging system.

### μCT analysis

Dissected femora were fixed in 4% paraformaldehyde, and analyzed using high-resolution μCT (SkyScan 1276). The femoral specimens were secured in a scanning tube with foam plates, adjusted to the length of each specimen. The scanner was configured to 65 kV and 200 μA at an 8 μm resolution. Image reconstruction software (NRecon), data analysis software (CTAn) and three-dimensional model visualization software (μCTVol V2.0) were utilized to analyze the distal femoral metaphyseal trabecular bone and the parameters of the diaphyseal cortical bone. Femoral cross-sectional images were generated to perform two-dimensional morphometric analysis of the cortical bone and three-dimensional morphometric analysis of the trabecular bone. The region of interest (ROI) was defined as the area extending from 5% of the femoral length proximal to the distal epiphyseal growth plate, extending proximally for a total of 5% of the femoral length. The trabecular bone volume fraction (Tb. BV/TV), trabecular thickness (Tb. Th), trabecular number (Tb. N) and trabecular separation (Tb. Sp) were analyzed. Additionally, a 20% to 30% ROI was selected to calculate the cortical bone volume fraction (Ct. BV/TV) and cortical thickness (Ct. Th). Rat femurs with implants were scanned and analyzed in a similar manner.

### Statistical analysis

Data were expressed as mean ± SEM. Data were analyzed with t test or one-way ANOVA using SPSS 16.0. Differences were considered statistically significant when *P* < 0.05.

## Results

### scRNA-seq profiling of the kidneys in DKD mice

**Figure 1.**
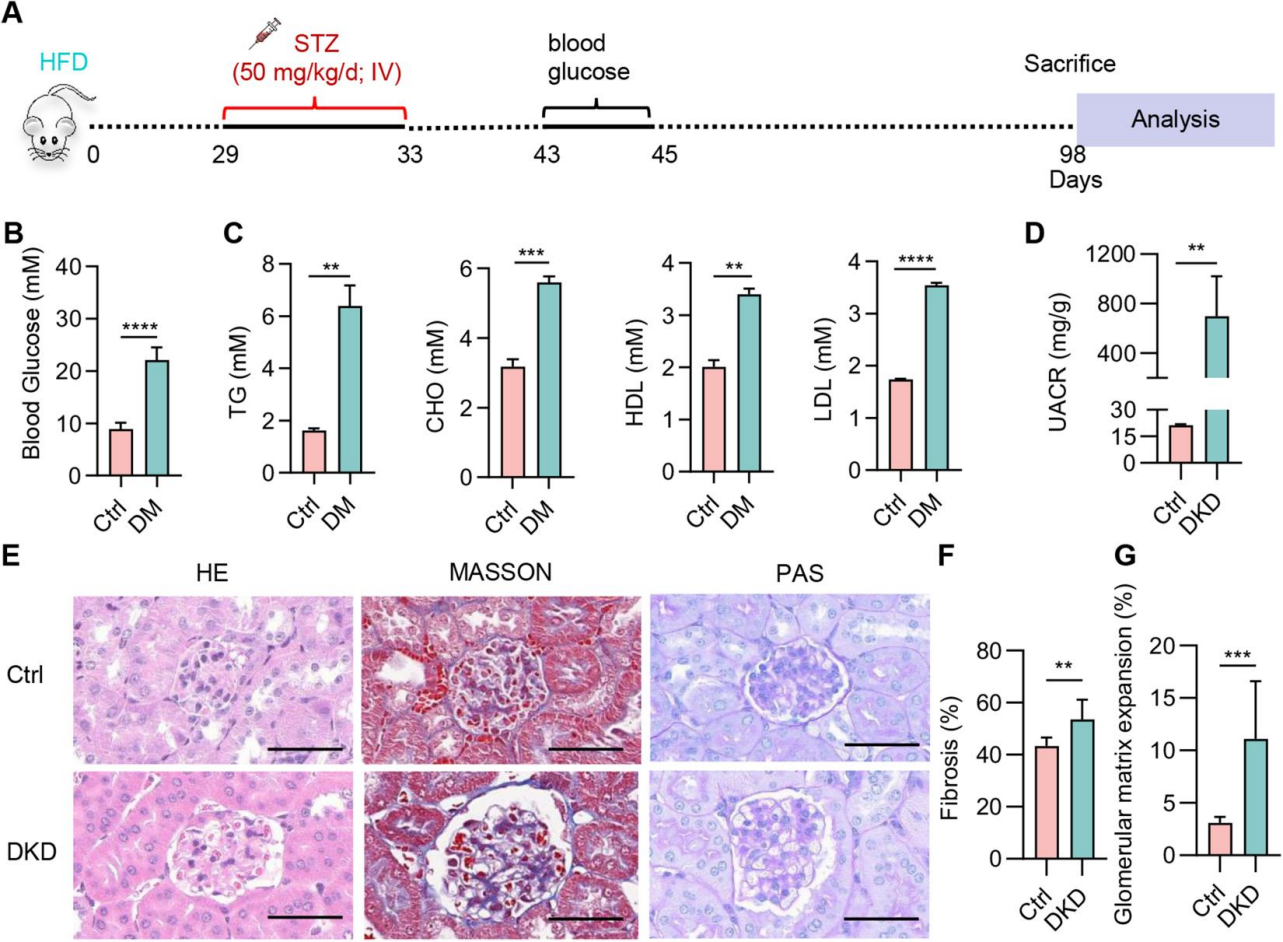
Establishment of diabetic kidney disease model in mice. (**A**) Experimental scheme. (**B**) Assessment of hyperglycemia in diabetic mice. (**C**) Levels of triglycerides (TG), cholesterol (CHO), high-density lipoprotein (HDL) and low-density lipoprotein (LDL) in the serum of mice. (**D**) Urinary albumin to creatinine ratio in control (Ctrl) and DKD group. (**E**) Representative image of H&E, Masson and PAS staining. Scale bar, 50 μm. (**F**) Quantification of the degree of fibrosis. (**G**) Quantification of mesangial matrix expansion. *n* = 6 per group. *P < 0.05. **P < 0.01. ***P < 0.001.

A diabetic mouse model was established by administering 50 mg/kg/day of STZ for five consecutive days to high-fat diet fed mice (Figure 1A). Both the diabetic and control mice were maintained on this diet for an additional eight weeks. In addition to elevated blood glucose levels and dyslipidemia (Figure 1B and C), the urinary albumin-creatinine ratio was significantly increased in the urine of diabetic mice (Figure 1D). To further assess kidney injury, we performed H&E, Masson’s trichrome and PAS staining. The results showed an increase in glomerular area, along with tubulointerstitial injury and mesangial expansion in the diabetic mice, confirming that these mice had progressed to the typical DKD stage (Figure 1E–G).

**Figure 2.**
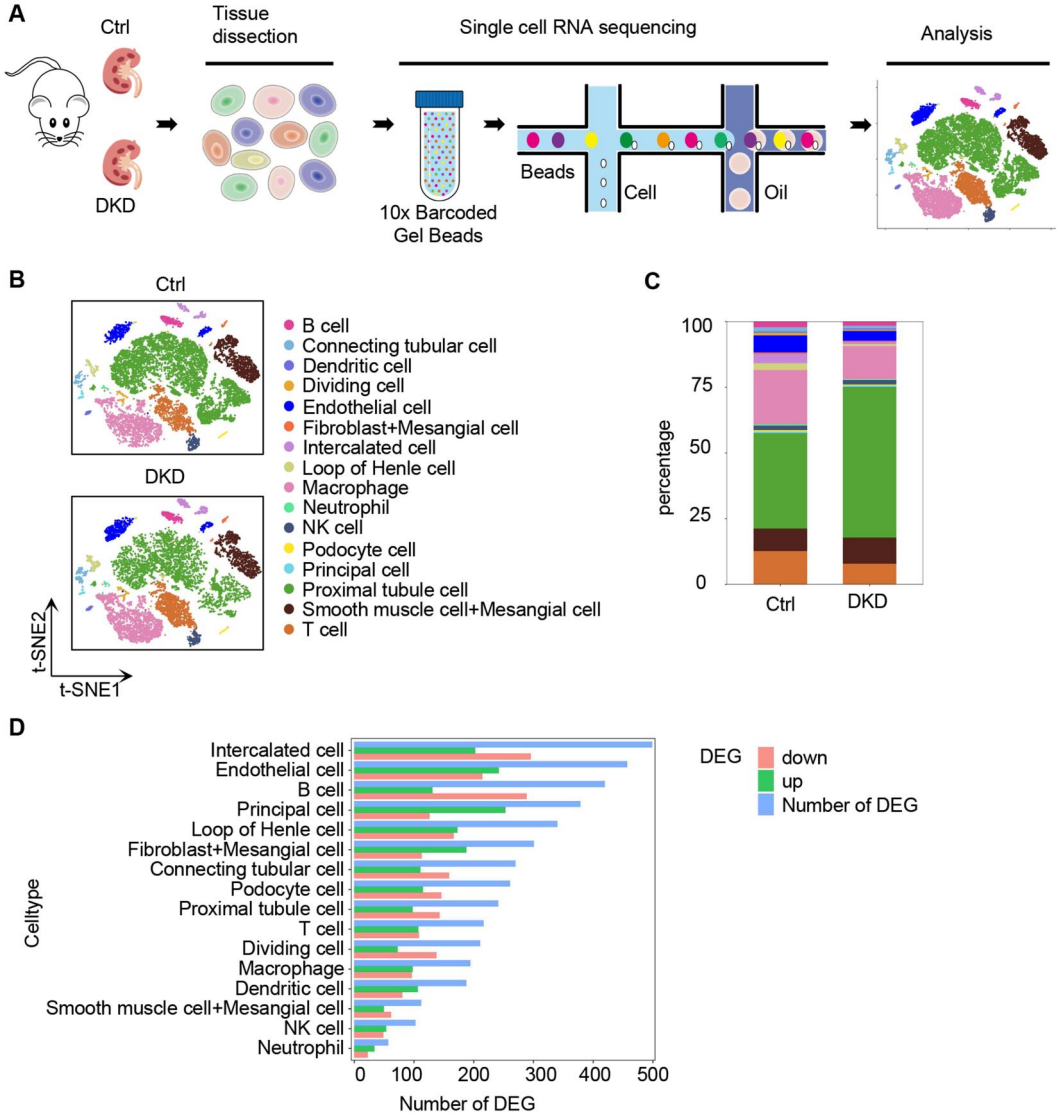
Unbiased clustering of scRNA-seq data revealing the cell type change in DKD. (**A**) Workflow for single-cell RNA transcriptome experiments. (**B**) t-SNE plot showing distinction of overall cell profiles in Ctrl and DKD groups. (**C**) Bar graph representing the frequency of cells acquired in each cluster. (**D**) Number of DE (differentially expressed) genes induced in DKD according to cell type.

Next, single-cell RNA sequencing (scRNA-seq) of kidney samples was performed on both control and DKD mice (Figure 2A). Six independent kidney samples were analyzed, comprising three from DKD mice and three from control mice. After tissue dissociation, 32 071 cells were obtained, and the cell viability was about 83%, ensuring the high quality of the data. Ultimately, a total of 23 055 high-quality cell sequencing data points were obtained. To visually illustrate the transcriptional differences among the cells, we employed t-distributed stochastic neighbor embedding (t-SNE), a dimensionality reduction technique, to project the cells into a two-dimensional space. This approach enabled us to successfully identify various major cell types in the kidney and their distribution patterns under disease conditions (Figure 2B). Based on cell-specific markers, we identified 31 distinct cell clusters belonging to 16 cell types, including B cells, connecting tubular cells, dendritic cells, dividing cells, endothelial cells, fibroblasts, intercalated cells, loop of Henle cells, macrophages, neutrophils, natural killer (NK) cells, podocytes, principal cells, proximal tubule cells, smooth muscle cells and T cells. MCs, which are key components of the glomerulus, play a crucial role in maintaining glomerular integrity. These cells share several markers with smooth muscle cells and fibroblasts [30, 31]. Thus, MCs were classified to either fibroblasts or smooth muscle cells due to the overlap of the markers. Proximal tubule cells and macrophages were the two predominant cell types in both the control and DKD kidneys (Figure 2C). Notably, intercalated cells, endothelial cells, B cells, principal cells and loop of Henle cells exhibited significant changes in gene expression, while immune cells, such as T cells, macrophages, dendritic cells, NK cells and neutrophils, showed smaller changes in gene expression (Figure 2D).

### Increased SFRP2 expression in MCs in the DKD

**Figure 3.**
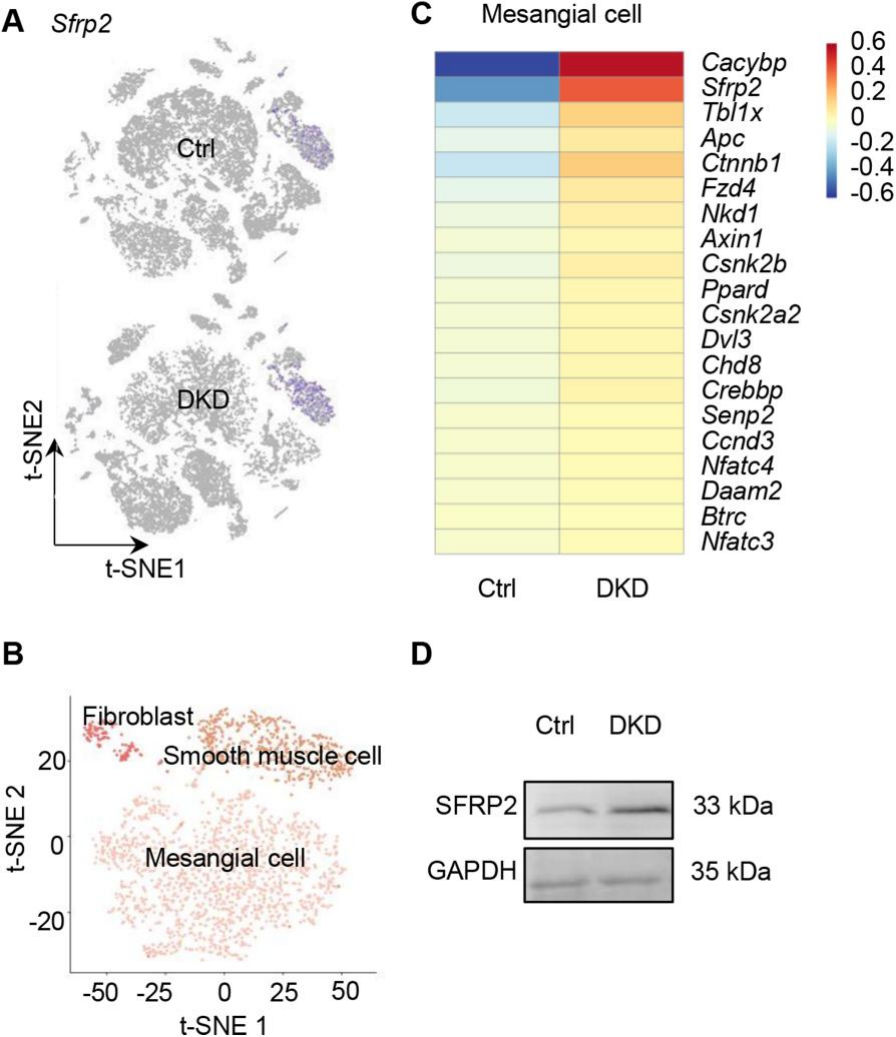
Increased SFRP2 expression in MCs in DKD. (**A**) Increased *Sfrp2* expression visualized in the t-SNE plot. (**B**) *Sfrp2*-expressing cells were found to be significantly enriched in smooth muscle cell and fibroblast clusters. (**C**) Heatmap showing the top 20 upregulated genes of Wnt pathway in MCs. (**D**) Western blot assay of SFRP2 in kidney tissues of control and DKD group.

Osteoporosis is one of the complications associated with diabetes [32]. Given the fundamental role of Wnt signaling in bone formation and repair, we investigated the expression profile of Wnt pathway in different cell types in the kidneys with DKD [33]. Wnt pathway was found mainly changed in PDGFR+ fibroblasts/smooth muscle cells/MCs and MCs are the dominant cell types in PDGFR+ cells (Figure 3A and B). Among the known Wnt regulators, *Sfrp2* was found to be the only secreted protein significantly increased in these cells (Figure 3A–C), highlighting the possibility involving in inter-organ communication. Accordingly, Western blot analysis demonstrated increased bulk expression of SFRP2 at the protein level in kidney samples from DKD group (Figure 3D).

### Exosomes efficiently deliver *siSfrp2* to glomerular MCs

**Figure 4.**
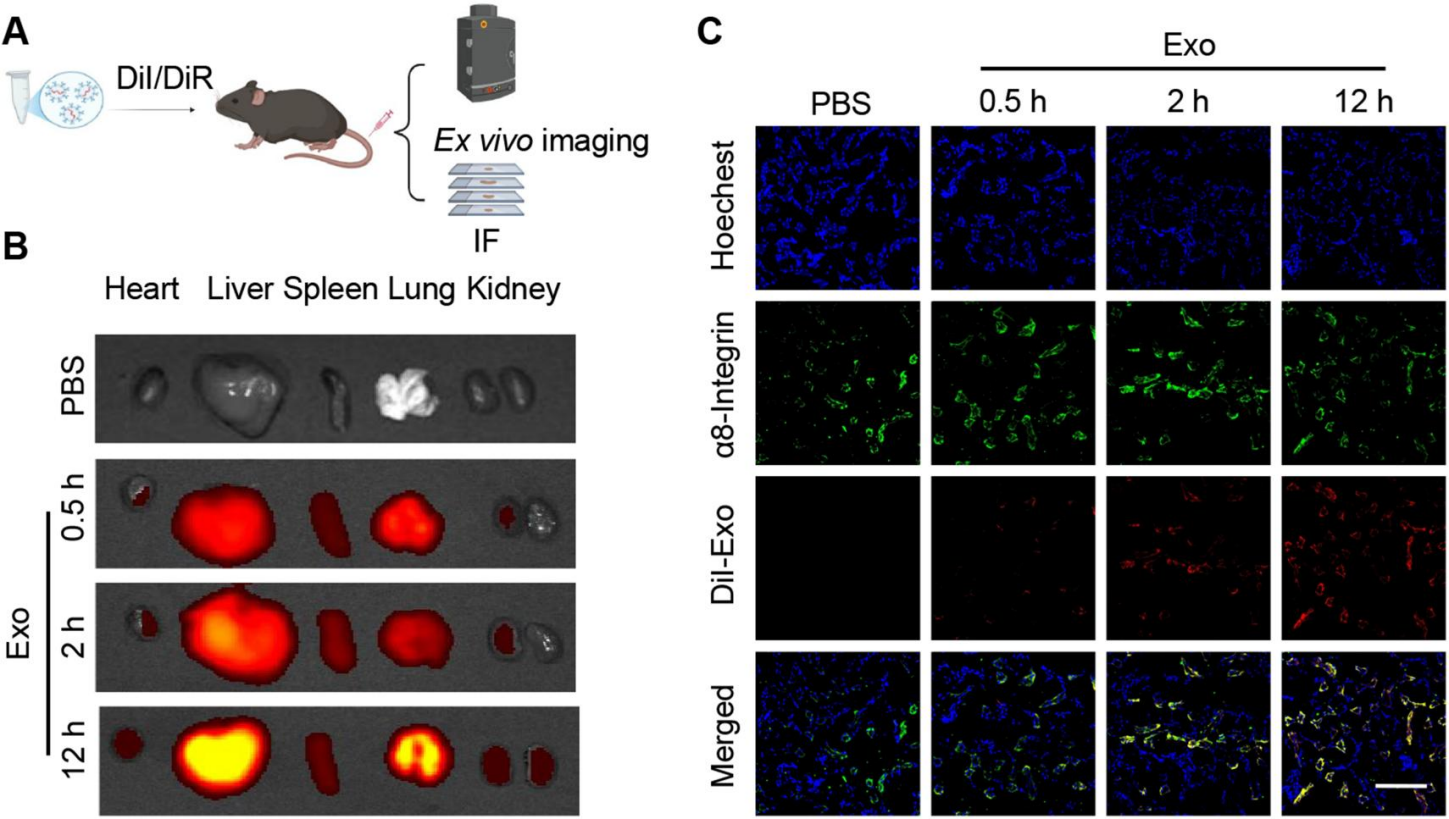
Targeted delivery of exosomes to the kidney. (**A**) Schematic representation of the experimental procedure. (**B**) *Ex vivo* fluorescence imaging demonstrates the fluorescence intensity of DiR-labeled EVs accumulated in the organs of C57BL/6J mice. (**C**) Immunofluorescence imaging shows the accumulation of DiI-labeled EVs in α8-integrin positive MCs of the kidney. Scale bars, 100 μm.

Nanoparticle size is a key determinant for deposition in the mesangial space [24]. Given the size range of exosomes, they might serve as an ideal vector for siRNA delivery into MCs. The exosomes isolated from HEK293T cells were characterized and identified using TEM, Western blotting and size distribution analysis (Supplementary Figure S1A–D). The particle size results indicate that over 50% of the exosomes had a diameter of less than 130 nm, which aligns with the requirements for deposition in the mesangial space. Both ex vivo fluorescence imaging and confocal microscopy analysis demonstrated that the liver, spleen and lungs were the primary target organs for exosomes (Figure 4A and B and Supplementary Figure S2). Notably, a significant number of exosomes were also delivered to the kidneys and were mainly accumulated within α8-integrin positive MCs (Figure 4C).

**Figure 5.**
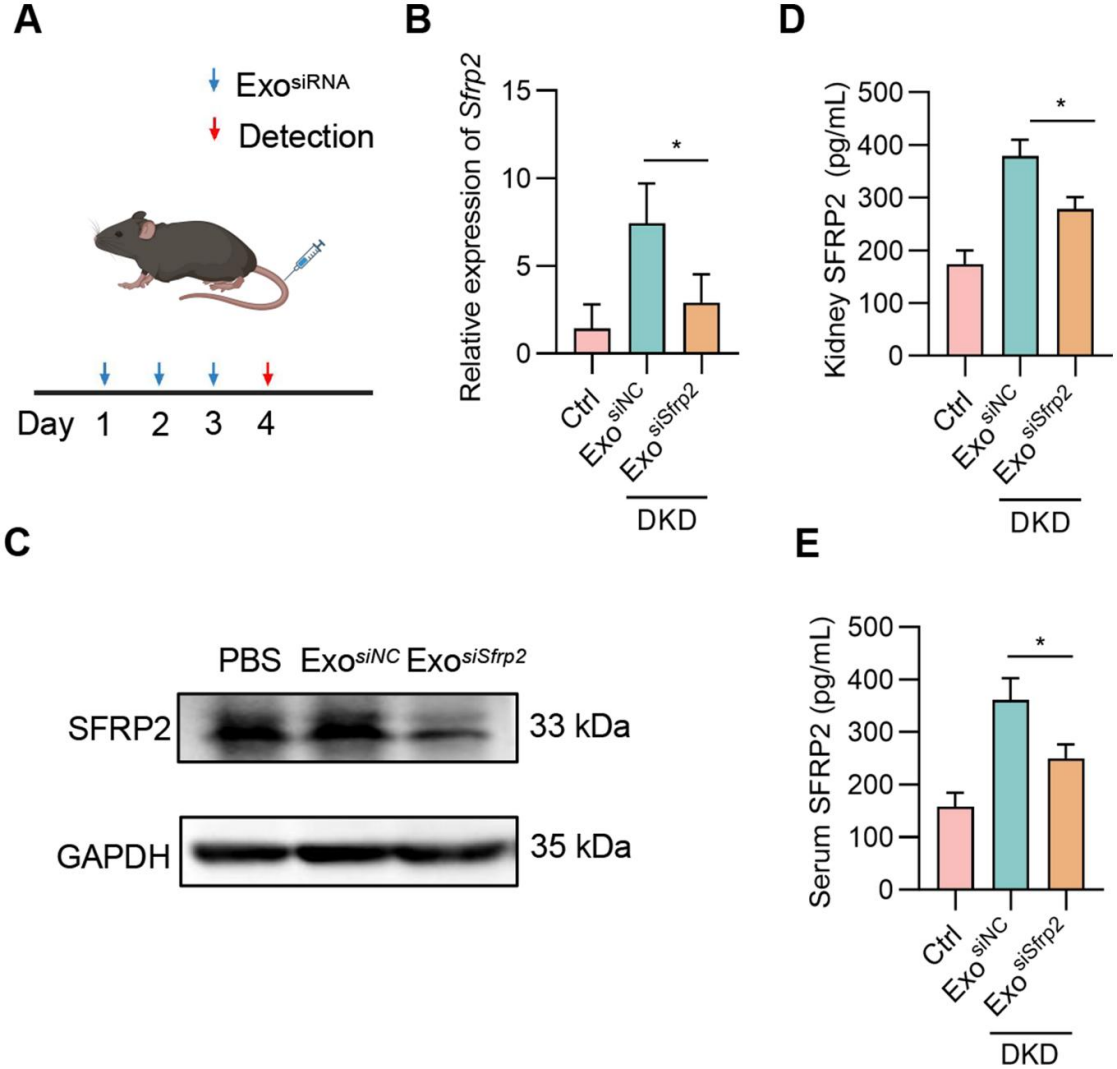
Exo^*siSfrp2*^ reduces SFRP2 in the kidneys of DKD mice. (**A**) Schematic representation of the experimental procedure. (**B**) Knockdown efficiency of Exo^*siSfrp2*^ in the kidney. (**C**) The protein expression level of SFRP2 in the kidney with indicated treatments. (**D**) The kidney secreted SFRP2 as analyzed by ELISA in mice with indicated treatments. A 100 mg kidney tissue sample was minced and then digested with collagenase in 1 mL PBS for 30 min, followed by ELISA analysis. (**E**) Serum SFRP2 as analyzed by ELISA in mice with indicated treatments. *n* = 3. *P < 0.05.

As anticipated, exosomes were efficiently loaded with *siSfrp2* via electroporation (Supplementary Figure S3A and B). Exosomes loaded with *siSfrp2* were denoted as Exo^*siSfrp2*^. *In vivo* delivery of the exosomes significantly reduced *Sfrp2* mRNA expression in the kidneys (Figure 5A and B). Consistent with the decreased *Sfrp2* mRNA expression in the kidneys, Western blot of renal lysis and ELISA analysis of the renal effusion revealed that SFRP2 expression at protein level was also found greatly reduced (Figure 5C and D). Moreover, serum SFRP2 was also found reduced after exosomal delivery of *siSfrp2* (Figure 5E). All of these data suggest that elevated SFRP2 from kidney in DKD may be at least one of the main sources of circulating SFRP2, while SFRP2 in organs including liver and spleen could not be excluded.

### Exosome-based knockdown of *Sfrp2* alleviates osteoporosis in DKD mice

**Figure 6.**
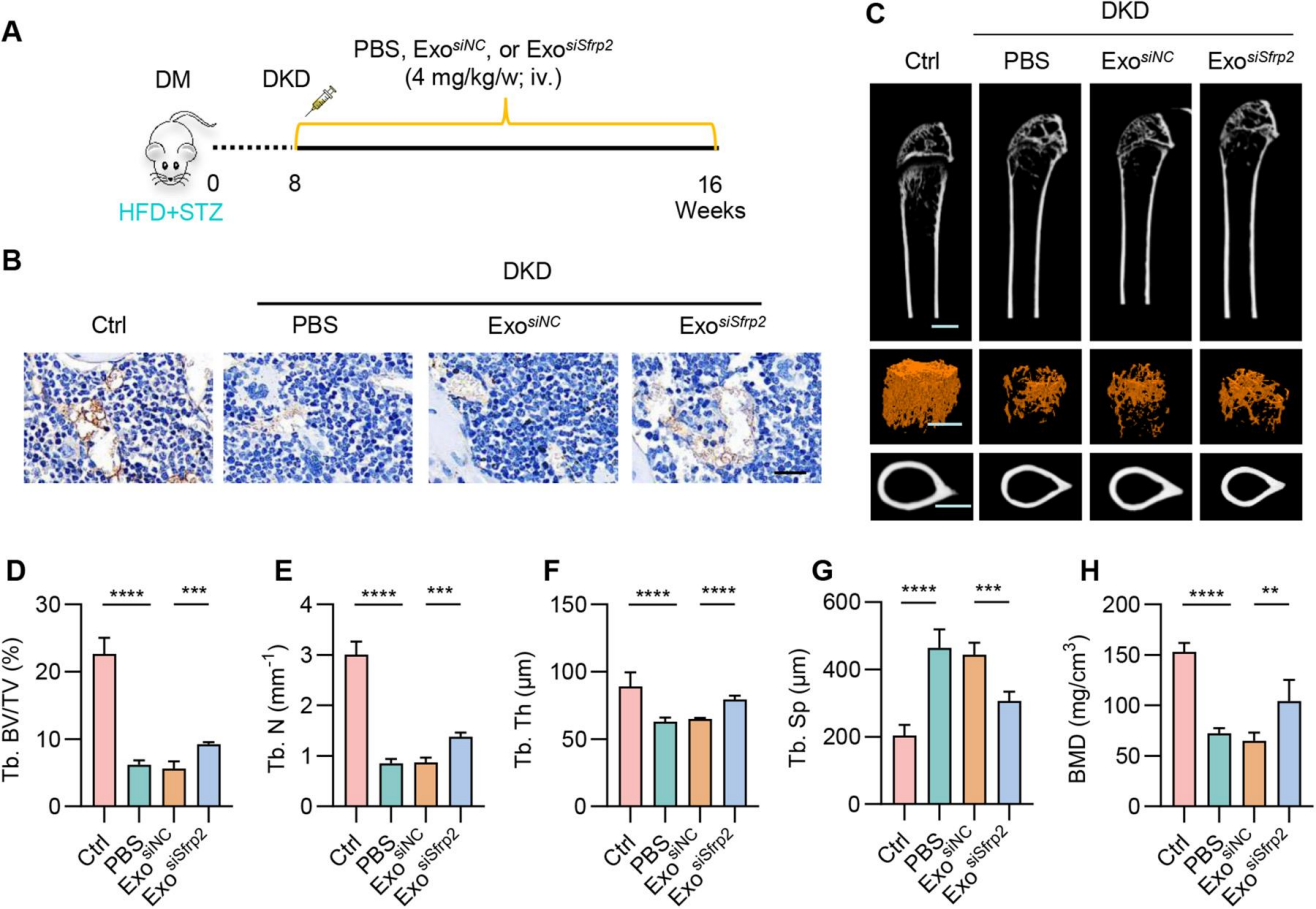
Exo^*siSfrp2*^ alleviates osteoporosis in DKD group. (**A**) Schematic representation of the experimental procedure. (**B**) The expression of β-catenin in bone marrow. Scale bar, 20 μm. (**C**) Representative μCT images of femora in indicated groups. Scale cars, 500 μm. (**D**–**G**) Quantitative μCT analysis of the Tb. BV/TV, Tb. N, Tb. Th and Tb. Sp in indicated groups. (**H**) The BMD of trabecular bone. *n* = 6. **P < 0.01. ***P < 0.001. ****P < 0.0001.

In the following experiments, we explored whether Exo^*siSfrp2*^ could alleviates osteoporosis in DKD group (Figure 6A). Immunohistochemistry analysis revealed that exosome-mediated *siSfrp2* delivery restored Wnt signal in the bone marrow of DKD mice, as shown by the increased β-catenin expression in bone marrow (Figure 6B). Consistent with the *in vivo* data, treatment with serum from DKD mice inhibited β-catenin nuclear translocation (Supplementary Figure S4A) and osteogenic gene expression (Supplementary Figure S4B–D) in MC-3T3E1 cells. Notably, the possible *Sfrp2* knockdown effects beyond kidneys, such as liver and spleen, might also be involved in the observed effects.

Consistent with previous findings that DKD mice display osteoporosis, μCT analysis of the femur revealed reduced bone mass and impaired microarchitecture in DKD group compared to the Ctrl group (Figure 6C). Consequently, the Tb. BV/TV, Tb. Th and Tb. N were lower, while Tb. Sp was higher in DKD mice (Figure 6D–G). Similarly, the bone mineral density (BMD) of trabecular bone in the DKD group was lower (Figure 6H). Strikingly, exosome-mediated *siSfrp2* delivery alleviates all the osteoporosis phenotype in DKD mice. Histological analyses of the heart, liver, spleen, lungs and kidneys from exosome-treated mice revealed that there were no obvious toxic effects found in mice treated with control or *siSfrp2*-loaded exosomes (Supplementary Figure S5), confirming the biosafety.

### Exosomal *siSfrp2* delivery enhances implant osseointegration in DKD rats

**Figure 7.**
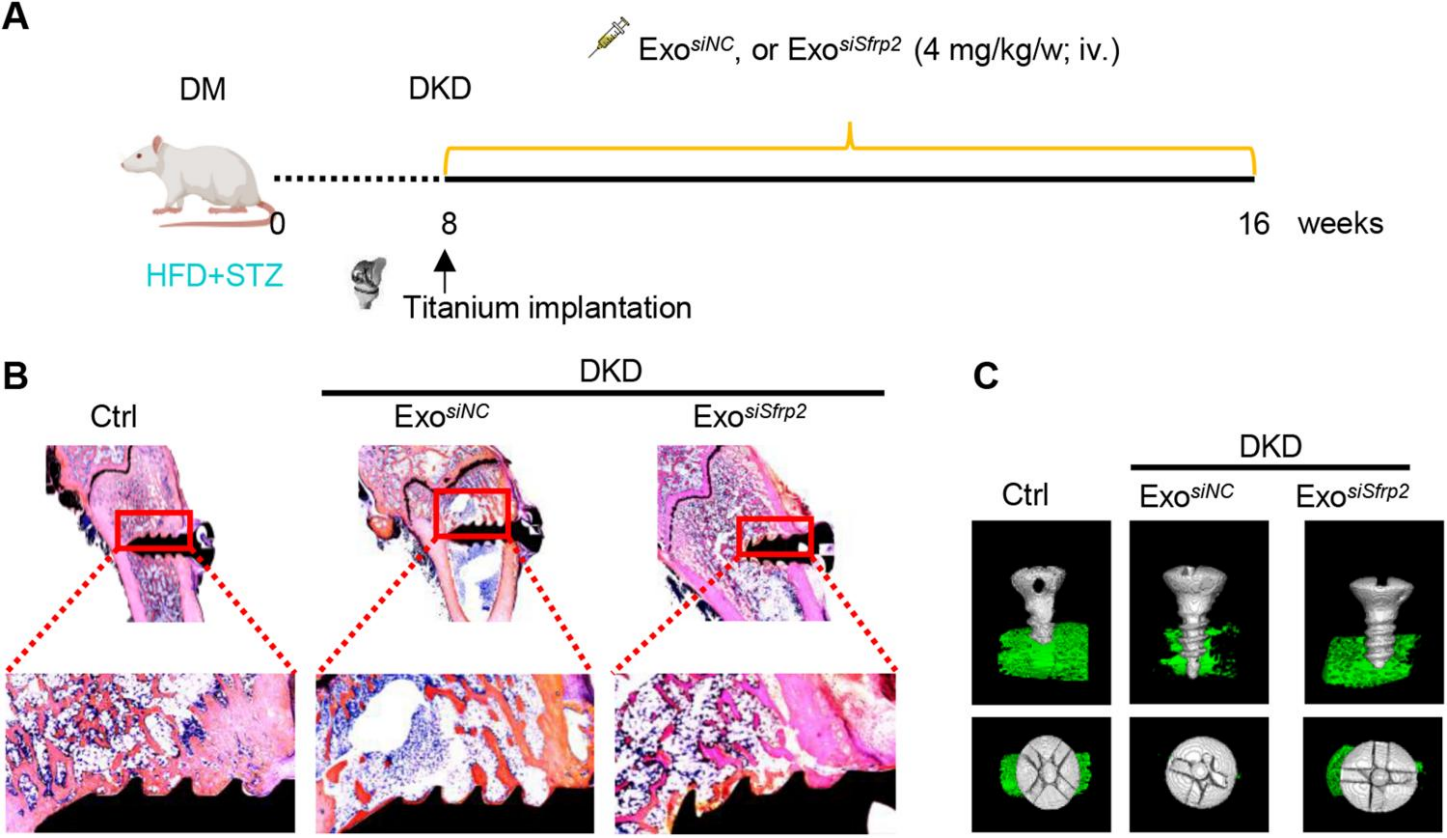
*siSfrp2* delivery via exosomes restores implant osteointegration. (**A**) Gross view of the implant. (**B**) Representative images of methylene blue/acid fuchsin staining showing that the new bone formed around the titanium implant after 8-week implantation. (**C**) Formation of new bone around the titanium implants based on 3-D reconstructed views of the micro-CT analysis 8 weeks post-implantation. *n* = 6.

Patients with DKD often experience impaired bone metabolism and delayed osseointegration following dental or orthopedic implant surgery, which presents a significant challenge for clinical treatment. In the subsequent experiments, we established a rat model of DKD using STZ injection in conjunction with a high-fat diet. After confirming the successful induction of DKD through measurement of blood glucose levels and renal function indices, including serum creatinine and blood urea nitrogen, we implanted titanium alloy implants into the tibiae of the DKD rats (Figure 7A). Age-matched normal rats served as controls. Consistent with findings in mice, exosomes effectively targeted MCs (Supplementary Figure S6) and exosome-mediated delivery of *siSfrp2* reversed the upregulation of *Sfrp2* in both the kidneys and serum in DKD rats (Supplementary Figure S7B–D). Micro-CT and histological staining analyses indicated a substantial reduction in implant osseointegration ability in DKD rats, while the reduction in *Sfrp2* significantly enhanced implant osseointegration in DKD rats, as demonstrated by micro-CT and histological analysis (Figure 7B and C).

## Discussion

Our study addresses a critical gap in understanding the renal-osteal crosstalk in diabetes by demonstrating that glomerular MCs serve as a major endocrine source of circulating secreted SFRP2 in DKD. Leveraging scRNA-seq of renal tissues, we identified MCs as the primary cellular source of SFRP2. Delivery of si*Sfrp2* into MCs with exosomes reduced serum SFRP2 levels *in vivo*, confirming their role as a key endocrine contributor to systemic SFRP2 elevation.

Clinically, this renal-osteal axis is pivotal in explaining the heightened osteoporosis risk in DKD. Epidemiological data from the referenced studies show that diabetic patients with DKD exhibit a significantly increased risk of low BMD and a strikingly higher fracture rate compared to those without kidney involvement [34–37]. Our mechanistic studies reveal that SFRP2 suppresses osteoblast differentiation by competitively inhibiting Wnt signaling. This mechanism differs from the age-related Klotho/FGF23 axis dysfunction described before [38–40], as it directly targets osteoblast commitment rather than mineral metabolism. Although we here proposed that MC-derived SFRP2 is a preliminary source of elevated serum level, it is highly possible that SFRP2 from other tissue/cell sources in addition to MCs might be also important, especially when considering that diabetes is a systemic disease and SFRP2 is widely expressed in multiple organs. Future experiments using MC-specific knockout model to confirm the detailed contribution of MC-derived SFRP2 are important. It is also important to explore whether elevated SFRP2 occurs in clinical patients. Previous studies revealed that a higher level of serum SFRP2 can be considered as an independent predictor of poorer clinical outcomes for elderly patients with acute exacerbation of chronic heart failure [41]. Whether SFRP2 serum level can serve as both a diagnostic indicator and a therapeutic target for DKD-related osteoporosis merits further investigation.

Our exosome-based therapeutic approach highlights the translational potential of targeting this axis. Combining SFRP2 inhibition with traditional anti-resorptive therapies (e.g. bisphosphonates) may address both defective bone formation (SFRP2-mediated) and enhanced resorption (inflammation-driven). However, challenges persist: the low payload capacity of exosomes and potential delivery efficiency decline in fibrotic DKD kidneys (where MC apoptosis may reduce target cell availability) require further optimization. Engineered exosomes functionalized with cell-targeting peptides (to enhance MC tropism) and microenvironment responsive capacity [42], thereby minimizing off-target effects in extrarenal tissues such as the liver and spleen, represents a promising strategy warranting further preclinical validation.

While our study establishes the MC-SFRP2-Wnt axis as a key driver of diabetic bone loss, several limitations warrant discussion. First, the temporal relationship between SFRP2 upregulation and glomerular fibrosis remains unclear; longitudinal studies are needed to determine if SFRP2 precedes or follows MC injury. Second, SFRP2 from other sources beyond kidneys could not be excluded. Notably, *siSfrp2*-loaded exosomes were distributed to organs beyond the kidneys and *Sfrp2* might also be expressed in these organs including liver and spleen. It is thus interesting to explore whether Sfrp2 from these organs also elevates and contributes to osteoporosis in DKD. Future studies to target delivery *siSfrp2* specific to kidney are necessary to confirm the detailed contribution of MC-derived SFRP2. Moreover, targeted knockdown of *Sfpr2* is also important to minimize side-effects concerning the physiological role of SFRP2 in these off-target organs. Lastly, the long-term safety of Wnt pathway modulation must be evaluated, given SFRP2’s potent role in non-skeletal tissues like the gut epithelium [43–45].

Notably, we here used HEK293-derived exosomes for mouse and rat models, and no obvious toxic effects were observed. Previous proteomic profiling analysis has revealed that even engineered exosomes from human sources (HEK293 cells) have rare immunogenicity in mice [46]. It could be explained by the conservative biogenesis process among species. In addition, the specific mechanisms for the uptake and processing of exosomes inside the cell might be immune-evasive. Together, our data here also strengthen the idea that HEK293 cell derived exosomes might be an ideal source with rare immunogenicity in the clinical context.

In conclusion, this work defines DKD as a critical driver in diabetic bone pathology, where MC-derived SFRP2 acts as a molecular bridge between renal inflammation and osteoblast dysfunction. By integrating scRNA-seq and functional exosome therapy, we provide mechanistic clarity to diabetes associated osteoporosis, and validate a precision therapeutic strategy targeting the renal-osteal axis. These findings emphasize the need to address organ crosstalk in context of diabetes-related osteoporosis.

## Supplementary Material

rbaf093_Supplementary_Data
