# Supplementary material for: Exosome-based Sfrp2 inhibition in mesangial cells alleviates osteoporosis and promotes osteointegration in diabetic kidney disease
Source: Regen Biomater. 2025 Sep 2;12:rbaf093. doi: 10.1093/rb/rbaf093 (PMC12478701; doi:10.1093/rb/rbaf093)
Supplement: rbaf093_Supplementary_Data [file rbaf093_supplementary_data.docx]

**Supplementary Information**

**Exosome-based Sfrp2 inhibition in mesangial cells alleviates osteoporosis and promotes osteointegration in diabetic kidney disease**

Helin Xing^1,#^, Yang Liu ^2,#^, Mi Qu ^2,3,#^, Zhengping Zhang ^4^, Yuhong Zeng^4^, Pan Li^5, *^, Qingsong Jiang ^1, *^, Guodong Yang^3,*^

**Table S1. siRNA sequences used in the study.**

| Name | sense | antisense |
| --- | --- | --- |
| *siSfrp2* | GCAUCGAGUACCAGAACAUTT | AUGUUCUGGUACUCGAUGCTT |
| *siNC* | UUCUCCGAACGUGUCACGUTT | ACGUGACACGUUCGGAGAATT |

**Table S2. Primers used in the study.**

| Gene | Forward primer | Reverse primer |
| --- | --- | --- |
| *Sfrp2* | GCCAGCCCGACTTCTCCTA | TAGCTCATGGTCTTGTACGCC |
| *Col1a1* | CCCTGGTCCCTCTGGAAATG | GGACCTTTGCCCCCTTCTTT |
| *Runx2* | ATCCCCATCCATCCACTCCA | AGTTCTGAAGCACCTGCCTG |
| *Alp* | GCTTTAAACCCAGACACAAG | AAGAAGAAGCCTTTGAGGTT |
| *Gapdh* | AGGTCGGTGTGAACGGATTTG | TGTAGACCATGTAGTTGAGGTCA |


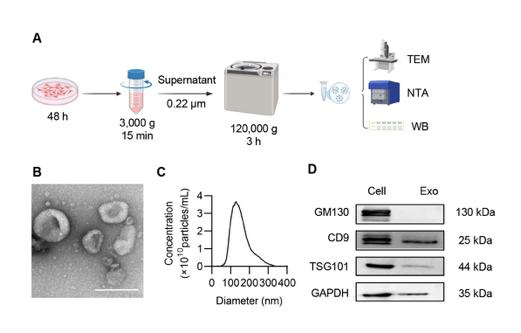


**Figure S1. Isolation and characterization of exosomes.** (A) Schematic representation of the experimental procedure. (B) Representative TEM images of exosomes. Scale bar, 200 nm. (C) Size distribution of the isolated exosomes. (D) Western blot analysis of the exosome markers CD9 and TSG101, the exclusive exosome marker GM130. GAPDH served as loading control.


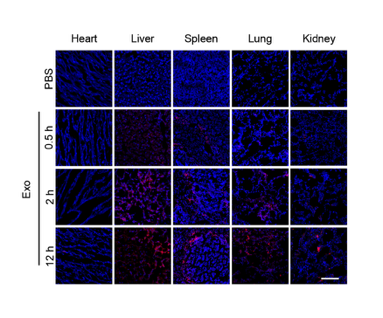


**Figure S2. Time-dependent enrichment of exosomes in kidney.**

Immunofluorescence imaging of DiR-labeled exosome distribution in various organs (heart, liver, spleen, lung, and kidney) at indicated time. Scale bar, 100 μm.


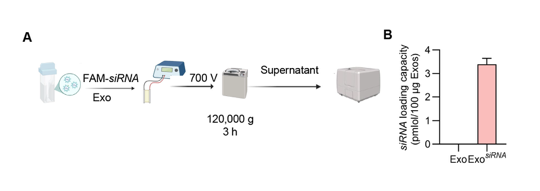


**Figure S3. Loading of *siRNA* into exosomes.** (A) Schematic representation of the experimental procedure for *siRNA* loading. (B) Loading efficiency of *siSfrp2* into exosomes. Data were expressed as mean±SEM. n=3.


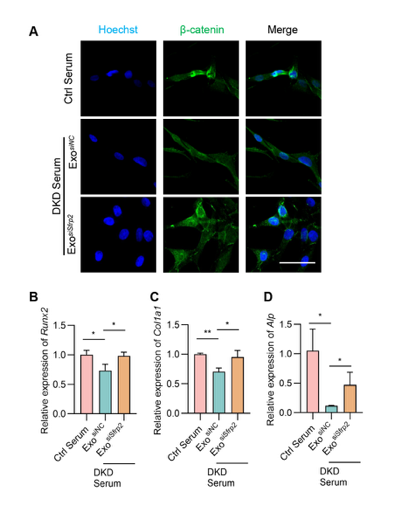


**Figure S4. Exo^siSfrp2^ treatment reverses the Wnt inhibitory function of DKD serum.** (A) MC-3T3E1 cells were incubated with serum from Ctrl or DKD mice with indicated treatments. Immunofluorescence images demonstrate the expression of β-catenin protein (green) in MC-3T3E1. Scale bar, 50 μm. (B-D) The mRNA expression level of *Runx2* (B), *Col1a1* (C) and *Alp* (D) in MC-3T3E1 treated same as above. n=3. *p$<$0.05. **p$<$0.01.


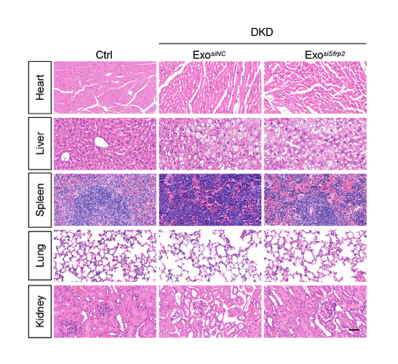


**Figure S5.** **H&E staining of indicated organs.** Scale bar, 50 μm.


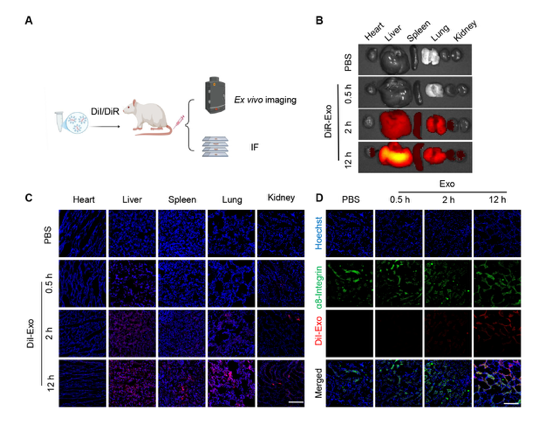


**Figure S6. *In vivo* distribution of injected exosomes of SD rats.** (A) Schematic representation of the experimental procedure. (B) *Ex vivo* fluorescence imaging of DiR-labeled exosome distribution in various organs (heart, liver, spleen, lung, and kidney) of SD rats. (C) Immunofluorescence imaging of DiR-labeled exosome distribution in various organs (heart, liver, spleen, lung, and kidney) of SD rats. Scale bar, 100 μm. (D) Immunofluorescence imaging shows the accumulation of DiI-labeled EVs (red) in mesangial cells (green) of SD rat kidneys. Scale bar, 100 μm.


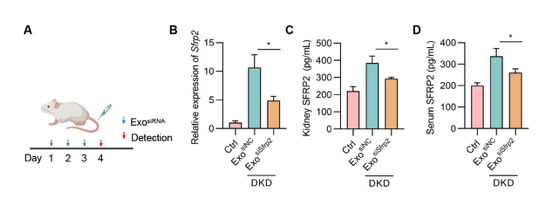


**Figure S7. Exo*^siSfrp2^* reduces *Sfrp2* in the kidney of DKD rat.** (A) Schematic representation of the experimental procedure. (B) Knockdown efficiency of *Sfrp2* in the rat kidney. (C) The kidney expression of SFRP2 as analyzed by ELISA in rats with indicated treatments. (D) The serum SFRP2 level as analyzed by ELISA in rats with indicated treatments. n=3. ^*^p$<$0.05.
